# Awareness and use of psychosocial care among cancer patients and their relatives—a comparison of people with and without a migration background in Germany

**DOI:** 10.1007/s00432-022-04091-1

**Published:** 2022-06-11

**Authors:** Susanne Singer, Nicola Riccetti, Isabelle Hempler, Marius Fried, Jorge Riera Knorrenschild, Louma Kalie, Martin Merbach, Marcel Reiser, Franz Mosthaf, Vitali Heidt, Kerstin Hermes-Moll

**Affiliations:** 1 Division of Epidemiology and Health Services Research, Institute of Medical Biostatistics, Epidemiology and Informatics (IMBEI), University Medical Centre, Johannes Gutenberg University, Obere Zahlbacher Straße 69, 55131 Mainz, Germany; 2 University Cancer Centre, Mainz, Germany; 3 Scientific Institute of Office-Based Haematologists and Oncologists (WINHO), Cologne, Germany; 4 Department of Palliative Medicine, University Medical Centre, Marburg-Gießen, Germany; 5 Institute of Pharmaceutical Sciences, Department of Pharmaceutical Technology and Biopharmacy, Albert-Ludwigs-University, Freiburg, Germany; 6 Central Institute for Family Counselling, Berlin, Germany; 7 Association of Binational Families and Couples, Berlin, Germany; 8 Community-Based Practice for Medical Oncology, Cologne, Germany; 9 Gemeinschaftspraxis für Hämatologie, Onkologie und Infektiologie, Zentrum für Ambulante Onkologie, Karlsruhe, Germany

**Keywords:** Cancer, Ethnicity, Healthcare disparities, Health services accessibility, Psycho-oncology, Vulnerable populations

## Abstract

**Purpose:**

We examined how migration background is associated with awareness and usage of psycho-oncology services.

**Methods:**

Oncologists in community-based practices and outpatient clinics asked their patients and their relatives to complete a questionnaire. Migrants were purposely over-sampled. The questionnaire was provided in Arabic, English, Farsi, French, German, Hindi, Kurdish, Pashto, Russian, Somali, Turkish, Urdu, and Vietnamese.

**Results:**

From 9 collaborators, 177 participants were enrolled (130 with and 47 without migration background). The existence of outpatient cancer counselling centres was known to 38% of the participants without and 32% with migration background, self-help groups to 32 vs. 12%, and psychotherapy to 43 vs. 25%. Respondents from the Near and Middle East were less likely to know about psychotherapy (odds ratio (OR) 0.1, *p* = 0.01); those from the Commonwealth of the Independent States or former Yugoslavia were less often informed about self-help groups (OR 0.1, *p* = 0.06). Migrants retrieved information less frequently from the internet than non-migrants (10 vs. 25%). At least one service had been used by 27% of migrants and 42% of non-migrants (OR 0.5, *p* = 0.06). After adjusting for gender, age, education, and patient-relative status, there was no evidence for an association between migration background and service use.

**Conclusions:**

Migrants should be better informed about psychotherapy and self-help groups, in particular the ones coming from the Near or Middle East and the Commonwealth of the Independent States or former Yugoslavia.

The under-use of psychosocial services can largely be explained by confounding factors. Therefore, these factors must always be taken into account when analysing the use of psychosocial services in the aforementioned populations.

**Supplementary Information:**

The online version contains supplementary material available at 10.1007/s00432-022-04091-1.

## Background

It is well known that people who have migrated to Western European countries use various types of health care—such as prevention, early detection, visits to general practitioners, rehabilitation, or community mental health care—less frequently than the autochthonous population, despite them having the same frequency and severity of diseases (Ahmad et al. 2021; Klein et al. 2018; Larchanche 2012; Wiessner et al. 2020; Zeeb et al. 2004). There are several reasons and mechanisms responsible for this under-use, for example being unaware of the existence of certain services (Larchanche 2012), language barriers (Fang and Baker 2013; Hyatt et al. 2018), not feeling entitled to use the services (Brenman 2021; Larchanche 2012), fear (Fang and Baker 2013), shame (Coleman-Brueckheimer et al. 2009; Fang and Baker 2013), a façading behaviour (Pergert 2017), misconceptions about treatment effects (James et al. 2011; Lourens 2013; Williams et al. 2019), or structural barriers such as financial problems and a lack of insurance (Fang and Baker 2013; Riccetti et al. 2022a, b). Often, it will be a mixture of several reasons.

It is not easy to pin down the “core causes” because migrants are not a homogeneous group of people. They have various cultural and personal backgrounds, different reasons for migration, and are in different phases of the migration process. The term “migrant” is in this respect misleading in its simplicity. Migration research faces the challenge of covering a breadth of aspects of migration—which is often done in large studies where all migrants are treated as one category (Zeeb et al. 2004; Zeissig et al. 2015)—and analysing the complexity of the phenomenon in-depth, often realised by including only certain groups, e.g. only individuals with a Russian or Turkish background, into the study (Cilenti et al. 2021; Erim et al. 2011; Graham et al. 2013; Morawa and Erim 2016; Ronellenfitsch et al. 2009). The first approach may deliver results that are too broad to effectively develop concrete suggestions for improvement of care, while the latter one might result in suggestions only relevant to certain groups of people. It was therefore our aim to find a balance between breadth and depth in our study investigating the use of psycho-oncological care by patients with a migrant background.

In Europe, only a few studies have investigated the care needs and treatment receipt of migrants in psycho-oncology (Ng et al. 2013; Weis et al. 2021; Zeissig et al. 2015). Most research on this topic stems from the United States or Canada (Riccetti et al. 2021) showing, for example, that African American and Hispanic women with cancer often report information needs regarding financial problems and not being able to provide for their families (Maguire et al. 2015), whereas this issue is less often mentioned by Chinese patients (Lim et al. 2017).

However, translating these findings to the European context is not directly possible because of the large differences in the health care systems.

Investigating the topic of psycho-oncology specifically seemed necessary because in some countries, mental health problems are severely stigmatised, resulting in the concealment of such problems and denial of the need to seek professional help (Saxena et al. 2007). Moreover, culture and religion can be associated with what people consider to be the cause of mental health problems (Lim et al. 2015; Sheikh and Furnham 2000), which in turn also influences the likelihood of using mental health care. For these reasons, it is possible that cancer patients and relatives with a migration background who experience mental health problems suffer from triple stigmatisation: being a member of an ethnic minority, having cancer, and suffering mentally. This may result in reduced use of psychosocial care (Merbach et al. 2006).

We, therefore, aimed to explore the awareness and the use of psycho-oncological services as well as specific treatment needs of immigrants from certain predefined regions of origin versus non-migrants in Germany.

More specifically, our research questions were:

1. What is the proportion of cancer patients and their relatives with and without a migration background who are informed about psychosocial services?
  - Where and when were they informed? What are their preferences in this regard?
  - In people with a migration background: In what language did they receive the information? What are their preferences in this regard? Do they know of such services from their country of origin?
2. What is the proportion of cancer patients and their relatives with and without a migration background who used psychosocial services?
3. Is awareness or usage of psycho-social services associated with characteristics of the migration background (i.e., region of origin, nationality, reason for migration, time since migration) and local language proficiency? Does this differ between patients and relatives?

## Methods

### Study design and data collection

This was a multi-centre, cross-sectional study in Germany. Cancer patients and their relatives were enrolled via a nationwide network of community-based practices specialising in medical oncology (Scientific Institute of Office-based Haematologists and Oncologists [“Wissenschaftliches Institut der Niedergelassenen Hämatologen und Onkologen”], WINHO) and outpatient cancer clinics at university hospitals. The doctors or nurses informed the patients and their relatives about the study and asked them to participate. They were reimbursed for this work.

The patients and relatives received an information leaflet and a questionnaire which was available in 13 different languages: Arabic, English, Farsi, French, German, Hindi, Kurdish, Pashto, Russian, Somali, Turkish, Urdu, and Vietnamese. These languages had been chosen because they are spoken by the majority of cancer patients with a non-German mother tongue (authors' calculations based on data from the WINHO patient survey 2015, unpublished) or by major migrant groups in Germany (Bundesamt 2016). If the questionnaire had not been translated into the native language of the participant, it was handed out in another language, mostly English, French, or German, depending on their preference.

The doctors were asked to approach individuals from different regions according to a predefined sampling matrix (see “Definition of the region of origin” section). We aimed at including a certain number of individuals per region. Unfortunately, this became difficult because of the COVID-19 pandemic, which began shortly after our study started (Hempler et al. 2021a, b, c), leading to more workload for the medical staff and more difficult logistics in meeting patients and relatives for interviews (which were necessary for the development of the questionnaire). We also included a sample of patients and relatives without a migration background for comparison purposes.

Further inclusion criteria were: a verified cancer diagnosis (all phases of disease) and a minimum age of 18 years. The only exclusion criterion was when the patient/relative was born outside Germany but both parents were of German nationality at that time.

The participants put the completed questionnaires anonymously into boxes that were provided by the collaborators. Because of that procedure, written consent was not necessary. It was not documented who participated and who did not, because it was assumed that ensuring the anonymity of the survey would increase the willingness to participate in the survey, especially in hard-to-reach populations (Enticott et al. 2017). Hence, the clinical characteristics of the participants are all self-reported.

The study protocol and conduct were supported by a patient representative with a migration background.

### Instrument

The questionnaire for this study was developed based on qualitative interviews with cancer patients who themselves or their parents had migrated to Germany and these patients’ relatives (Hempler et al. 2021a, b, c) and on qualitative interviews with cancer specialists (Hempler et al. 2021a, b, c). We asked about the following:

*Characteristics of the migration background:* country of birth, country of mother’s birth, country of father’s birth, year of migration, reason(s) for migration (e.g., refuge/asylum seeker/international protection, work, marriage).

*German language proficiency:* This was ascertained by asking whether the doctor-patient consultations need to be translated.

*Awareness of services:* We asked whether they were informed about cancer counselling centres, self-help groups, psychotherapy, and other types of support (with the possibility to write down what other types of support), and where they had been informed, and at what time during the course of treatment. Individuals who were born outside of Germany were asked whether they knew of such services from their country of origin. They were also asked in what language they had received the information and whether they had wished to receive it in another language.

*Use of psychosocial services:* Respondents indicated whether they had used cancer counselling centres, self-help groups, psychotherapy, and/or other types of support.

*Emotional and social functioning* were ascertained using the respective scales of the European Organization for Research and Treatment of Cancer Core Instrument (EORTC QLQ-C30) (Aaronson et al. 1993). Items were summarised and transformed to scores between 0 and 100. Higher scores indicate better functioning. Patients were grouped into “high distress” if their functioning was worse than the threshold of clinical importance (Giesinger et al. 2020).

### Data analysis

#### Definition of a migration background

Individuals were defined as “having a migration background” if one or more of the following criteria were fulfilled:

- they had been born outside of Germany
- one or both of their parents were born outside of Germany
- their nationality was not German or both German and non-German (dual citizen)
- their mother tongue was not German

Further, we ascertained the generation of the migrants (first, second, or third generation).

#### Definition of the region of origin

The pre-defined regions of origin were: Near and Middle East (including North Africa); Sub-Saharan Africa; Southeast Asia and East Asia; Commonwealth of Independent States (including Ukraine and Georgia), the Baltic States, and former Yugoslavia (including Albania); European Union (except former Yugoslavia and the Baltic States) or North America (USA, Canada); all other countries. Individuals were coded as “coming from this region” if they or their parents were born in a country in this particular region. If patient and parents came from different regions, the region of the parents was used to define the region of origin. If mother and father came from different regions, the region of the mother was used.

If the country of birth was unknown, the language of the questionnaire was used as a proxy to define the region if it was sufficiently clear. For example, if the questionnaire was in Russian, the region was coded as “Commonwealth of Independent States”. If, however, the questionnaire was in English and no other information about the country of birth or mother tongue was available, the region was left as “unknown”.

#### Statistical approach

Group differences between participants with and without a migration background were explored with chi-square and *t*-tests. Then, the association of characteristics of the migration background (i.e., region of origin, nationality, reason for migration, time since migration) and German language proficiency with awareness and use of psychosocial services were investigated using binary logistic regression analyses while adjusting for gender, age, and education. Emotional and social functioning were not adjusted for because they were considered to be on the causal pathway.

We report the odds ratios (OR) and use 95% confidence intervals to quantify statistical uncertainty.

Potential effect modification by patient-relative status was explored using Mantel–Haenszel tests and likelihood-ratio tests.

Statistical analyses were performed using STATA 15 (StataCorp 2017, College Station, TX: StataCorp LP).

## Results

### Sample

In total, 184 completed questionnaires were collected during the study period (June to September 2021). In 7 of them, the migration status could not be clearly classified, leaving 177 for the analysis. Among them, 130 had a migration background and 47 did not (for details see Table 1). The regions of origin were primarily the Near and the Middle East and the Commonwealth of Independent States. No patient from Sub-Saharan Africa was enrolled.

**Table 1:** Respondents’ demographic and clinical characteristics

	Total (*n* = 177)		Without Migration Background (*n* = 47)		With Migration Background (*n* = 130)	
	*N*	%	*N*	%	*N*	%
Gender						
Male	67	38%	12	26%	55	42%
Female	98	55%	35	74%	63	48%
Unknown	12	7%	0	0%	12	9%
Age						
Mean (min–max)	58.7	(20–86)	62.6	(37–86)	57.0	(20–85)
Relative						
Patient	152	86%	46	98%	106	82%
Relative	25	14%	1	2%	24	18%
Education						
University	36	20%	11	23%	25	19%
Vocational education	33	19%	7	15%	26	20%
College	20	11%	5	11%	15	12%
Post-compulsory	38	21%	17	36%	21	16%
Compulsory	26	15%	7	15%	19	15%
Other	2	1%	0	0%	2	2%
None	7	4%	0	0%	7	5%
Unknown	15	8%	0	0%	15	12%
Practice vs. Clinic						
Community-based practice	112	63%	36	77%	76	58%
Outpatient clinic	65	37%	11	23%	54	42%
In those with a migration background						
Nationality						
Non-German only					41	32%
German and Non-German					4	3%
German only					49	38%
Unknown					36	28%
Region of origin						
Near and Middle East					44	34%
(South-)East Asia					9	7%
Commonwealth of the Independent States, the Baltic States, and Ex-Yugoslavia					40	31%
Other outside of EU and outside North America					5	4%
EU (but not Germany) or North America					13	10%
Germany					8	6%
Not further specified					11	8%
Year of migration						
Mean (min–max)					1990	(1949–2021)
Generation						
First generation					117	90%
Second generation					5	4%
Third generation					3	2%
Unknown					5	4%
Reason for migration						
Refuge/asylum seeker					9	7%
Work					14	11%
Education					4	3%
Family reunion					28	22%
Creation of a family					2	2%
Wish to stay in Germany					8	6%
Other					8	6%
Unknown					57	44%
Language proficiency						
Translation of consultation necessary					57	44%

Participants with and without a migration background differed in many demographic aspects (Table 1), which is why these variables were taken into account when comparing the groups regarding awareness and use of psychosocial services.

Among the participants with a migration background, 117 (90%) were first-generation migrants (Table 1).

### Emotional and social functioning

Among participants from all regions, emotional and social functioning were below the means of the general population (Supplemental Material, eFigure 1). Participants with a migration background had an average score of 60.3 in emotional functioning, while non-migrants had a mean score of 53.2 (*p* = 0.18). The percentage of self-reported increased emotional distress was 57% in migrants and 72% in non-migrants (*p* = 0.07).

Social functioning was on average better in migrants than in non-migrants (58.5 versus 41.5, *p* = 0.005). The proportion of self-reported increased social distress was 47% among migrants and 60% among non-migrants (*p* = 0.14).

### Awareness of psychosocial services

Among the participants, 38% of non-migrants and 32% of migrants reported being aware of cancer counselling centres (*p* = 0.04), 32 and 12% of self-help groups (*p* = 0.002), 43 and 25% of psychotherapy (*p* = 0.03), and 13 and 8% of other support (*p* = 0.21). Other support options mentioned included: consultation-liaison services, social services at the hospital, general practitioners, and staff of health care insurance. The number of services known ranged from 0 to 4, with an average of 1.3 (in those without migration: 1.5, in those with migration: 1.2, *p* = 0.04). At least one of the services was known to 87% of the respondents without and 86% with a migration background (OR 0.89, *p* = 0.85).

Of the respondents who were born outside of Germany (*n* = 107), 7 (7%) said they knew about such services in their country of origin, 85 (79%) said there were none, and 15 (14%) did not reply to this question. Notably, none of the patients/relatives from (South-) East Asia and from other non-EU/non-North American countries knew about such services in their country of origin, while 10% (*n* = 4) of the ones from the Commonwealth of Independent States did.

### Sources of information

The majority of the respondents received information about services in the hospital and/or community-based practices (Table 2). The internet (10 vs. 25%, *p* = 0.02) and flyers (6 vs. 15%, *p* = 0.08) were less often mentioned as an information source by patients and relatives with a migration background. They also generally had fewer other sources of information compared to non-migrants (1 vs. 10%, *p* = 0.01).

**Table 2:** Sources of information about psychosocial services

Where did you receive information about psychosocial support services?	Percentage among respondents *without* migration background	Percentage among respondents *with* migration background	*p*-value
In the hospital	55	51	0.63
In this practice	43	45	0.83
In another practice	10	12	0.75
From friends and family	35	26	0.27
During rehabilitation	3	5	0.52
In a cancer counselling centre	3	8	0.24
In a patient self-help group	0	3	0.27
In the internet	25	10	0.02
Via flyers	15	6	0.08
Other sources of information	10	1	0.01

### Preferred and actual time-point of information

**Fig. 1:**
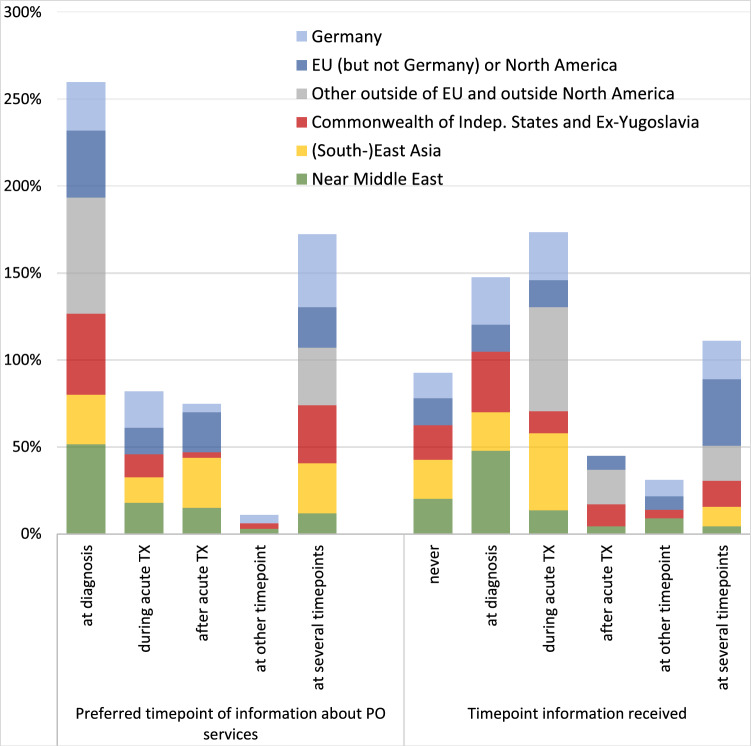
Preferred and actual time-point for receipt of information about psychosocial services, by region of origin. *Notes *Displayed are the cumulative percentages per region, thereby balancing out the different sample sizes per region

The most often preferred time-point for information about psychosocial services was at the time of diagnosis and at several time points during the disease trajectory (Fig. 1). They most frequently received this information during acute treatment.

There were few differences between respondents from different regions regarding preferences. For example, people from the Near and Middle East as well as those from other countries outside of the EU and North America more often expressed the wish to receive information already at diagnosis, whereas individuals from East and Southeast Asia less often did so (Fig. 1).

### Language of information material

Among the migrants (*n* = 130), the majority (64%) had received information about psychosocial services in German only, 10% in another language, 8% both in German and another language, and 18% did not answer this question.

The other languages were Turkish (*n* = 9), Russian (*n* = 4), English (*n* = 2) as well as Arabic, Ukrainian, Urdu, Serbian/Bosnian/Croatian (each with *n* = 1), or not further specified (*n* = 5).

A total of 17 (13%) said they had wished they had received information in another language, 68 (52%) were satisfied with the language they had received, and 45 (35%) did not answer this question. The most frequently mentioned language in which patients hat wished to receive information was Russian (*n* = 11), followed by Dutch (*n* = 2), Polish, Bulgarian, Lithuanian, and Indonesian (each with *n* = 1).

### Use of psychosocial services

The most frequently used psychosocial service both in migrants and non-migrants was psychotherapy (12 and 28%, respectively, *p* = 0.01). Outpatient cancer counselling centres had been visited by 8% of migrants and 4% of non-migrants (*p* = 0.42), self-help groups by 1% of migrants and 9% of non-migrants (*p* = 0.01), other support by 5% of migrants and 15% of non-migrants (*p* = 0.04). The sum of services used ranged from none to 4, with an average of 0.3 in migrants and 0.6 in non-migrants (*p* = 0.01).

At least one service was used by 27% of migrants and 42% of non-migrants (odds ratio 0.5, *p* = 0.06).

### Association of migration background characteristics with awareness and use of psychosocial services

We found no effect modifications by patient-relative status; hence, we included it as a potential confounder in the models. Reasons for migration and time since migration had too many missing values and were therefore excluded. As self-help groups were only used by 5 respondents, we used the combined use of psychosocial services as the outcome variable (either cancer counselling service or psychotherapy or self-help group or other service or a combination of these).

When adjusting for age, gender, and education, there were only a few associations of migration background characteristics with awareness and usage of psychosocial services (Table 3): respondents from the European Union or North America had 11 times the odds of being informed about cancer counselling centres; those from the Near and Middle East were less likely to be informed about psychotherapy; those from the Commonwealth of the Independent States, the Baltic States or former Yugoslavia were less often informed about self-help groups.

**Table 3:** Association of migration background with being aware of and using psychosocial services

		Is informed about…									Uses …		
		Cancer Counselling Centres			Psychotherapy			Self-help groups			Any service		
		OR	95%CI	*p*	OR	95%CI	*p*	OR	95%CI	*p*	OR	95%CI	*p*
Region	Germany	Ref			Ref			Ref			Ref		
	Near Middle East	1.0	(0.3–4.0)	0.97	0.1	(0.0–0.5)	0.01	0.8	(0.2–3.8)	0.74	0.3	(0.1–1.5)	0.15
	(South-)East Asia	3.9	(0.4–36.6)	0.23	Omitted (no one was informed about psychotherapy)			Omitted (no one was informed about self-help groups)			1.2	(0.1–13.4)	0.89
	Commonwealth of Independent States, Baltic States, and Ex-Yugoslavia	1.8	(0.5–6.0)	0.35	0.4	(0.1–1.6)	0.19	0.1	(0.0–1.1)	0.06	1.1	(0.3–4.3)	0.92
	Other outside of EU and outside North America	0.4	(0.0–4.3)	0.45	0.8	(0.1–7.9)	0.89	1.0	(0.1–12.0)	1.00	1.7	(0.2–14.6)	0.63
	EU (but not Germany) or North America	11.3	(1.7–76.4)	0.01	0.6	(0.1–3.3)	0.58	2.6	(0.5–13.2)	0.26	0.6	(0.1–3.6)	0.56
Nationality	German (vs. non-German)	0.6	(0.2–2.1)	0.45	0.7	(0.2–2.9)	0.67	1.0	(0.2–5.4)	0.98	2.7	(0.7–10.5)	0.16
Language Proficiency	Needs translation (vs. needs no translation)	0.5	(0.1–1.7)	0.24	3.2	(0.7–14.8)	0.13	1.5	(0.3–8.9)	0.65	1.8	(0.4–8.1)	0.44
Relative vs. Patient	Respondent is a relative (vs. a patient)	0.4	(0.1–2.4)	0.29	0.0	(0.0–0.6)	0.02	Omitted (no one was informed about self-help groups)			0.4	(0.0–2.7)	0.32

All estimates are adjusted for age, gender, and education

*OR *Odds Ratio, *CI *Confidence Interval, *p *p-value, *Ref *Reference, *EU *European Union

Women had 2.4 times the chance of being informed about cancer counselling centres (*p* = 0.08), whereas no differences in awareness about other services were present. Being informed about psychotherapy was negatively correlated with the age of the responders (OR 0.9, *p* < 0.01). Compared to responders with compulsory education only, the ones with post-compulsory training (OR 0.2, *p* = 0.01) and a university degree (OR 0.2, *p* = 0.02) were less likely to be aware of psychotherapy.

Use of psychosocial services was less likely among people from the Near and Middle East and more likely in those with German nationality, though the confidence intervals were large in both cases (Table 3). Women were more likely to use psychosocial services compared to men (OR 2.7, *p* = 0.07), and with increasing age, the odds of using services decreased (OR 0.9, *p* = 0.05). There was no evidence for an independent effect of education on psychosocial services use.

## Discussion

With this study, we examined how cancer patients (and their relatives) with a migration background are informed about psychosocial services and how often they make use of it compared to non-migrants.

A recent study from the UK (Ahmad et al. 2021) found that the prevalence of common mental disorders did not differ between ethnic groups if socioeconomic and demographic characteristics were taken into account. Treatment receipt, however, did. Asian, non-British white, and especially black people received treatment less often (i.e., antidepressant medication, counselling or therapy, talking to a general practitioner for mental, nervous or emotional complaint, visiting a community mental health specialist in the past 12 months). Even more concerning is the finding that these inequalities increased between 2007 and 2014 despite the “Improving Access to Psychological Therapies (IAPT)” programme that was launched there in 2008 to decrease inequalities in mental health care.

We also found considerable differences regarding awareness and usage of psycho-oncological services. However, some of them were likely due to confounding factors. For example, the proportion of men was higher in migrants than in non-migrants. It is well known that men use psychosocial services less often than women (Bayer et al. 2020; Doherty and Kartalova-O'doherty 2010; Oliffe and Phillips 2008; Plakun 2021) , and this effect was present in our study, too. Hence, part of the differences between migrants and non-migrants in the use of psychosocial services can be explained by gender. This again underlines the notion that it is not enough to provide special services to “migrants in general”, but we must identify the vulnerable groups among them; we must ask what groups of patients and relatives need what type of support.

Still, even when taking gender, age, education, and relative status into account, in our study, people coming from the Near and Middle East were clearly less likely to be informed about psychotherapy and hence less able to use these services, which is in line with the results of Ahmad et al. (2021). As this result was also adjusted for German language proficiency, it is unlikely that this under-information is solely due to communication barriers. Possible reasons for this result could be related to lacking cultural sensibility on the side of health care providers or underlying cultural/religious beliefs not captured in this study on the side of patients.

Another relevant point was that in the group of migrants, the proportion of relatives was higher than in the non-migrants, and relatives were also less frequently informed about the various services. This is in line with other research showing that relatives are less often aware that they are entitled to use psycho-oncological services (Billaudelle et al. 2022; Meyer et al. 2015; Singer et al. 2022).

The limited power of our study is another explanation for this “loss” of association when adjusting for other variables and looking in more detail at which factors of the migration background might play a role. Our aim had been to include about 50 patients per region, but this became difficult due to the COVID-19 pandemic. For example, relatives were not allowed to accompany patients anymore, making it more difficult to hand out the questionnaires to them. In addition, the doctors in the practices were barely able to perform their usual daily workload and could not approach as many patients as they usually would (Hempler et al. 2021a, b, c). Colleagues from two outpatient clinics offered to help and indeed enrolled about a third of all participants. However, the study period could not be extended due to the end of funding and so some cells in the sampling matrix remained empty (for people from Sub-Saharan Africa) or filled with fewer than 50 individuals. This is a clear limitation of our study.

A positive feature is that we were able to enrol quite a few people from the Near and Middle East and the Commonwealth of Independent States. We could provide the questionnaire in several languages which is considered to be good practice to increase participation in studies with migrants (Reiss et al. 2013). By this, we were able to reduce the selection bias due to missing language proficiency. Our results also underline that patients and relatives often prefer to receive information in their native language. This is not only necessary for the “transfer of knowledge” in people with limited language proficiency it also gives a sense of familiarity in a situation of increased insecurity, which is also important for those who perfectly speak the language of the health care professional (Hempler et al. 2021a, b, c). Another aspect can be related to being able to speak in the native language: In many cultures, the women are responsible for taking care of the family’s health. Hence, using the mother tongue can be related to being cared for and feeling comforted (Arghavanian et al. 2020; Graham et al. 1985; Mesler et al. 2022; Zhang et al. 2019).

Research found that to ease access for migrants, health care not only needs translation of consultations or information material but also culturally sensitive approaches and openness to the “otherness” of the patients (Merbach 2019; Röhnsch and Flick 2015; Schrank et al. 2017). In a multi-centre randomised controlled trial, Hölzel et al. showed that information material was evaluated to be more useful by patients of Russian, Turkish, Polish, and Italian origin who had depression or chronic low back pain if it was culturally sensitive in contrast to a simple translation (Holzel et al. 2016). This effect was larger in patients with low levels of acculturation.

Hence, translating information material about psychosocial services and adapting it culturally for the various migrant groups seems very valuable. However, our study also showed the challenges of such an endeavour. Instead of one set of questionnaires, we sent 13 sets to our collaborators—one in each language. This needs to be stored somewhere in the clinic or practice. A possible solution would be to provide this material online. However, we found that the migrants in our study used the internet less often than non-migrants; it would therefore be advisable that the doctor or nurse prints the information out and hands it to the patients and relatives.

Another relevant aspect is the doctor–patient-relationship. Our interviews and survey of doctors indicate that they experience issues with the doctor–patient-relationship more often with patients from the Near or Middle East, Sub-Saharan Africa, and the Commonwealth of Independent States (Hempler et al. 2021a, b, c; Hermes-Moll et al. 2022; Riccetti et al. 2022a, b). The doctors themselves often related these problems in the relationship to language barriers. However, it is also possible that other factors, more or less unconsciously, influence the way they communicate with patients coming from countries or cultures they feel unfamiliar with. For example, they may expect that patients from specific countries or of a certain gender are not interested in taking up psychotherapy due to stereotypes and thus do not provide such information. As a consequence, some patients may be left less well informed about the services they could use. In combination with the patient’s own feelings of not being entitled to use services (Brenman 2021; Larchanche 2012), fears (Fang et al. 2013), or feelings of shame (Coleman-Brueckheimer et al. 2009; Fang et al. 2013), this can lead to psycho-oncological under-care for these patients. To counter such processes, it might be advisable to set up a standard procedure where all patients and relatives, independent of their ethnic or religious background, are asked about their need for support and are informed about available services—not only that they exist but also what they can offer and how to access them (Hermes-Moll et al. 2022).

An important limitation of our study is that respondents from Southeast and East Asia and from Sub-Saharan Africa could not be enrolled as often as we had hoped, resulting in small numbers and making it more difficult to compare awareness and usage of psychosocial services in these groups with other groups.

Another problem was that not all participants completed the crucial questions about migration background due to a mistake (logical inconsistency) in the questionnaire. As the data collection was anonymised, they could not be contacted and health care staff could not complete this information. This further reduced the sample size (7 questionnaires had to be excluded).

### Clinical implications

Cancer patients and their relatives who have a migration background are not as well informed about psychosocial services as non-migrants. Only 12% of them use psychotherapy compared to 28% of patients and relatives without a migration background. These differences can largely be explained by differences in age, gender, and education, which are in turn related to the awareness and use of psychosocial services. To support cancer patients and their relatives, clinicians should provide information about psycho-oncological services at the time of diagnosis as well as during the course of the treatment, optimally in the patients’ native language and culturally adapted. Moreover, information should be provided in a standardized way, so that everybody is equally informed, independent of their age, gender, education, religion, culture, languages spoken, etc. This is already a requirement for hospitals to be certified by the German Cancer Society (Herschbach and Mandel 2011; Kowalski et al. 2016) and is a recommendation for the outpatient sector, too (Hermes-Moll et al. 2022).

Psychosocial problems and care needs should also be ascertained using instruments in the language the patient prefers. The instruments could be stored electronically in multiple languages and then be printed out when needed.

Further, more and better training of medical staff in culturally sensitive counselling is needed. This should already start during university, but must be continued over the entire span of their career.

How the services can be provided in their mother tongue remains an open question. It requires further research and probably the development of new, innovative programs.

The establishment of culturally specific patient support groups in corresponding languages might also be helpful.

### Conclusions

Cancer patients and their relatives with a migration background should receive more information about psychotherapy and self-help groups in particular. This is especially true for people coming from the Near or Middle East regarding information about psychotherapy and for people from the Commonwealth of the Independent States or former Yugoslavia regarding information about self-help groups.

Migrants also use psychosocial services less often than non-migrants. However, this difference can largely be explained by confounding factors such as gender, age, education, and being a relative versus a patient. These factors must therefore always be taken into account when analysing service use in migrant populations.

## Supplementary Information

Below is the link to the electronic supplementary material.

Supplementary file1 (DOCX 69 KB)

## Data Availability

The data that support the findings of this study are available on reasonable request from the corresponding author. The data are not publicly available due to privacy restrictions.
