# Supplementary material for: Awareness and use of psychosocial care among cancer patients and their relatives—a comparison of people with and without a migration background in Germany
Source: J Cancer Res Clin Oncol. 2022 Jun 11;149(5):1733–45. doi: 10.1007/s00432-022-04091-1 (PMC9188276; doi:10.1007/s00432-022-04091-1)
Supplement: Supplementary file 1 — Supplementary file1 (DOCX 69 KB) [file 432_2022_4091_MOESM1_ESM.docx]

# Electronic material


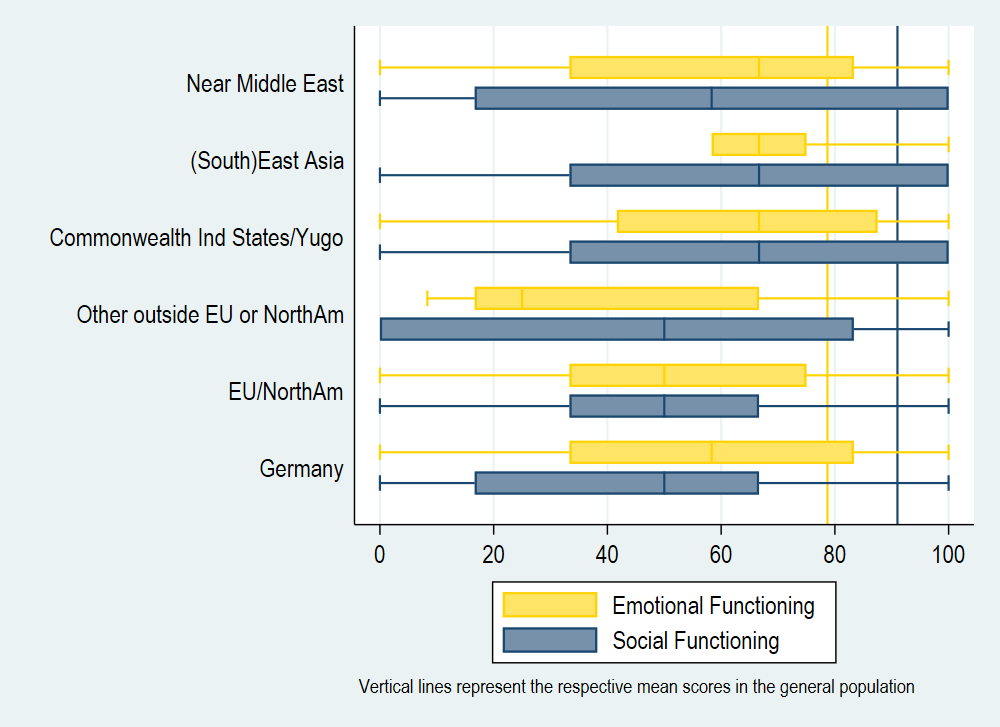


eFigure 1 Emotional and Social Functioning, by region of origin

| *Notes* |  | Higher scores indicate better functioning, i.e., less problems. |
| --- | --- | --- |
|  | NorthAm… | North America (USA, Canada) |
|  | EU … | European Union |
|  | Yugo… | Former Yugoslavia |
